# Cutaneous Horn-Related Kaposi's Sarcoma: A Case Report

**DOI:** 10.1155/2010/825949

**Published:** 2010-08-30

**Authors:** Nilufer Onak Kandemir, Banu Dogan Gun, Figen Barut, Nilgun Solak Tekin, Sukru Oguz Ozdamar

**Affiliations:** ^1^Department of Pathology, Faculty of Medicine, Zonguldak Karaelmas University, 67100 Zonguldak, Turkey; ^2^Department of Dermathology, Faculty of Medicine, Zonguldak Karaelmas University, 67100 Zonguldak, Turkey

## Abstract

Cutaneous horn is characterized by the accumulation of abnormal keratinized material and may occur in association with a variety of benign, premalignant, and malignant cutaneous lesions. Cutaneous horn occurs very rarely in association with soft-tissue neoplasias. A cutaneous horn located on the toe was completely removed by excision in a 78-year-old male patient. Macroscopic examination revealed a hemorrhagic nodular lesion, 0.5 cm in diameter, located on the dermis underlying the cutaneous horn with a height of 1 cm. Histopathological examination revealed a neoplastic lesion consisting of fusiform cells and extravasated erythrocytes underlying the compact keratin mass. The immunohistochemical analysis showed immunoexpression of endothelial markers and HHV8 in fusiform cells. The case was evaluated as “cutaneous horn developed in a nodular stage Kaposi's sarcoma.” Our case is the second case of cutaneous horn related to Kaposi's sarcoma reported in the English literature and is presented in this case report with its clinical and histopathological features.

## 1. Introduction

Cutaneous horns (cornu cutaneum) are chronic, dense hyperkeratotic cutaneous protrusions that often resemble the horn of an animal. Cutaneous horns are found in the upper parts of the body such as face, neck, and shoulders and are frequently related to actinic damages [1]. Even though 60% of the cutaneous lesions underlying compact keratin layer are benign, malignant, or premalignant, lesions might be associated with it. Bowen's disease, squamous cell carcinoma, basal cell carcinoma, and malignant melanoma are the most frequent malignancies seen on the base of cutaneous horns [2]. 

Cutaneous horn occurs very rarely in association with soft-tissue neoplasias [3]. In this paper, a cutaneous horn case arising from a classical KS base located on the foot has been presented as it is a rare clinical finding.

## 2. Case

A 78-year-old male patient with a diagnosis of KS who has been followed for three years presented with a rapidly growing, brown, horn-like lesion between the second and third toes of his right foot that appeared 2 months prior. The hyperkeratotic lesion that was 0,5 cm in diameter and 1 cm in length had been totally removed by excision with a clinical prediagnosis of cornu cutaneum. Three years before the current diagnosis, the patient underwent excision of pink-to-purple, slightly protuberant lesions on the plantar surface of the left foot and on the pretibial area. After the histopathological examination a KS diagnosis in patch and nodule stage was given. HIV test (enzyme-linked immunosorbent assay and Western blot) was negative. The patient's family history remained insignificant; however the personal past medical history revealed that the patient had been treated for hypertension and insulin-dependant diabetes mellitus. The patient did not receive radiotherapy or chemotherapy during the period he had been followed for KS and his routine biochemical examination was unremarkable. He had got only cutaneous involvement by KS; mucosal and systemic disease had not been detected. 

**Figure 1:**
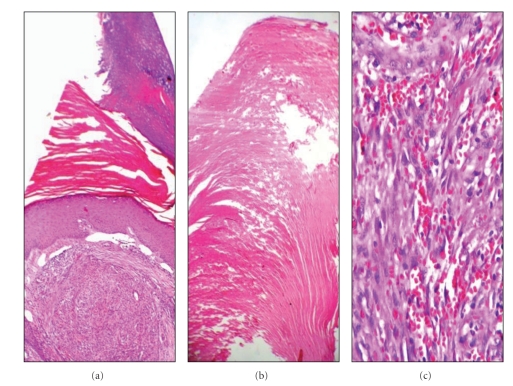
(a) Nodular stage Kaposi's sarcoma containing compact keratin layer in the epidermis (Hematoxylin-Eosin [H-E], x40). (b) Lamellar keratin accumulation on the epidermis at the surface of the lesion (H-E, x100). (c) Neoplastic proliferation consisting of fusiform cells and extravasated erythrocytes in nodular stage Kaposi's sarcoma (H-E, x200).

**Figure 2:**
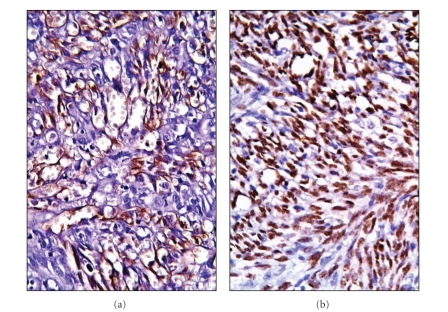
CD31 (a) and HHV-8 (b) immunoreactivity in neoplastic spindle cells ((a)-(b): AEC-DAB, x400).

 Histopathological examination revealed a nodular tumour tissue under the hyperkeratotic epidermis, separated from surrounding tissue with irregular borders, consisting of fusiform cells, extravasated erythrocytes, and irregular vascular structures. Rare atypical mitosis and cytoplasmic hyaline globules were noted in the tumor. A cutaneous horn structure composed of compact keratin was observed on the epidermis at the tumour surface (Figures 1(a)–1(c)). The immunohistochemical analysis showed a positive cytoplasmic reaction with lymphovascular endothelial markers (CD31, CD34, and D2-40) and a positive nuclear reaction with human herpesvirus-8, latent nuclear antigen-1 (HHV-8, LNA-1) in the neoplastic cells (Figure 2). In line with these histopathological and immunohistochemical data, the case was evaluated as “cutaneous horn developed in a nodular stage Kaposi's sarcoma base”. Six months after the operation, no recurrence was noted. The patient is not presently under treatment for Kaposi's sarcoma. 

## 3. Discussion

Cutaneous horn is a morphologic designation referring to cohesive keratinized material protuberant above the surface of the skin but not a true pathologic entity. The pathogenesis of this abnormal formation of keratinized material has not been fully elucidated. It may be of clinical importance because the underlying condition may be a malignant lesion. It is difficult to define the underlying lesion, especially in superficial biopsies. Therefore, deep (punch) biopsies or total excision of small lesions are recommended to be certain, and a specimen from the lesion under the keratin layer is obtained [1–3]. Cutaneous horn occurs most frequently in association with benign and malignant lesions of epidermis and cutaneous appendages. Extremely rare cases associated with metastatic renal cell carcinoma [4], lymphoma [5], dermatofibroma [6], and pyogenic granuloma [7] have been reported. Among the predisposed factors, advanced age, male sex, and sun exposure have importance [3]. 

To the best of our knowledge, there is only one previous report of cutaneous horn related to Kaposi's sarcoma in the literature [8]. Histological subtypes of KS include a hyperkeratotic variant. Hyperkeratotic KS is usually described in KS cases associated with AIDS and can develop on a chronic lymphedema base. In this variant, there is marked hyperkeratosis, acanthosis, and papillomatosis overlying the epidermis of the lesion. However, compact keratin accumulation, specific to cutaneous horn, is not seen [9]. In our case, nodular stage Kaposi's sarcoma has been found at the base of cutaneous horn; typical horn structure containing compact keratin may be seen at the surface. 

 Histological features including acanthosis and papillomatosis typical for hyperkeratotic KS are not present in the epidermis, and the horn structure is clinically visible at the surface of the lesion. Another feature of our case is that the cutaneous horn is located on the toe, which is an unexpected area for these types of lesions. This suggests that trauma might be a predisposing factor for the development of cutaneous horn in areas that are not exposed to actinic damage. In addition, the advanced age and gender of our case support the role of the age and sex in the aetiology of cutaneous horn development, as reported in the literature. In conclusion, although it is rare, cutaneous horn may be associated with soft-tissue neoplasms. Adequate and appropriate surgical sampling is important for the determination of the underlying lesion.
